# Insights into the antioxidant, anti-inflammatory and anti-microbial potential of *Nigella sativa* essential oil against oral pathogens

**DOI:** 10.1038/s41598-024-62915-1

**Published:** 2024-05-24

**Authors:** Shaeesta Khaleelahmed Bhavikatti, Siti Lailatul Akmar Zainuddin, Rosmaliza Binti Ramli, Sameer J. Nadaf, Padma B. Dandge, Masidd Khalate, Mohmed Isaqali Karobari

**Affiliations:** 1 Department of Periodontics, School of Dental Sciences, Universiti Sains Malaysia, Health Campus, Kubang Kerian, 16150 Kelantan, Malaysia; 2 Department of Dental Research, Centre for Global Health Research, Saveetha Medical College and Hospitals, Saveetha Institute of Medical and Technical Sciences, Chennai, 602105 Tamil Nadu India; 3 Faculty of Dentistry, Manipal University College Malaysia, Jalan Batu Hampar, Bukit Baru, 75150 Melaka, Malaysia; 4 Basic and Medical Sciences Unit, School of Dental Sciences, Health Campus, Universiti Sains Malaysia, Kota Bharu16150, Kelantan, Malaysia; 5 Bharati Vidyapeeth College of Pharmacy, Palus, 416310 Maharashtra India; 6 Department of Biochemistry, Shivaji University, Kolhapur, 416004 Maharastra India; 7 Department of Biotechnology, Shivaji University, Kolhapur, 416004 Maharastra India; 8 Department of Restorative Dentistry and Endodontics, Faculty of Dentistry, University of Puthisastra, Phnom Penh, 12211 Cambodia

**Keywords:** Dental, *Nigella sativa*, Oral health, Phytochemicals, Hydro-distillation, Oral pathogens, Periodontitis, Oral microbiology, Periodontics

## Abstract

Oral disorders can exert systemic ramifications beyond their localized effects on dental tissues, implicating a wide array of physiological conditions. The utilization of essential oils (EOs) for protection of oral health represents a longstanding practice. Consequently, in this investigation, essential oil derived from *Nigella sativa* seeds (NSEO) underwent isolation via the hydro-distillation process, followed by a comprehensive evaluation of its antioxidant, anti-inflammatory, anti-fungal, antibacterial activities, and cytocompatibility. The isolated NSEO manifested as a pale-yellow substance and was found to harbor a diverse spectrum of bioactive constituents, including steroids, triterpenoids, flavonoids, phenols, proteins, alkaloids, tannin, sesquiterpenoid hydrocarbons, monoterpenoid alcohol, and monoterpenoid ketone (thymoquinone). Notably, the total phenolic content (TPC) and total flavonoid content (TFC) of NSEO were quantified at 641.23 μg GAE/gm and 442.25 μg QE/g, respectively. Furthermore, NSEO exhibited concentration-dependent inhibition of protein denaturation, HRBC membrane stabilization, and hemolysis inhibition. Comparative analysis revealed that NSEO and chlorhexidine (CHX) 0.2% displayed substantial inhibition of hemolysis compared to aspirin. While NSEO and CHX 0.2% demonstrated analogous antibacterial activity against *Escherichia coli*, *Staphylococcus aureus*, and *Pseudomonas aeruginosa*, NSEO showcased heightened efficacy against *Lactobacillus acidophilus* and *Candida albicans*. Additionally, NSEO exhibited pronounced effects against periodontal pathogens such as *Aggregatibacter actinomycetemcomitans*, *Porphyromonas gingivalis*, *Tannerella forsythia*, and *Prevotella intermedia*. Importantly, no cytotoxicity was observed on human gingival fibroblast cell lines. These findings underscore the potential of NSEO as a potent antibacterial and antifungal agent in the management of oral microbial pathogens, thereby offering avenues for the development of innovative therapies targeting diverse oral inflammatory conditions. Nevertheless, further investigations are imperative to unlock its full therapeutic repertoire.

## Introduction

Oral health is a crucial aspect of well-being and possesses a notable influence on systemic health. Improper dental wellness is associated with an increased risk of preterm birth, gastrointestinal problems in older people, cardiac disease, infectious endocarditis, and bacteria-related pneumonia^1,2^.

Periodontitis is an inflammatory condition that affects the periodontium, the tissues that support teeth. Global Burden of Disease Study (2016) reveals that, severe periodontal disease ranked as the 11th most common condition globally. Nearly 20–50% of people worldwide have periodontal disease, primarily in its mild and moderate forms, whereas 10% of adults worldwide have periodontal disease in its chronic form, which grows especially during the 3rd and 4th decades of life^3,4^. Subgingival plaque bacteria, particularly those of the "red complex" group i.e. *Porphyromonas gingivalis*, *Tannerella forsythia*, and *Treponema denticola*, are the primary etiologic agents of periodontitis. Apart from the red complex, bacteria belonging to, *Aggregatibacter actinomycetemcomitans P. gingivalis* and *T. denticola* species also contribute to periodontitis^5^. Periodontal treatment endeavors to establish a root surface that meets biological acceptability criteria by eradicating pathogenic bacteria and their associated metabolites. In periodontal treatment, several antibiotics are used as adjunct to the conventional therapy for periodontitis. However, the rise of multidrug-resistant bacteria and cytotoxicity to human gingival fibroblast (HGF) cells poses a significant challenge, leading to treatment failures. Thus, developing antimicrobial drugs that prevent drug resistance emergence with minimum cytotoxicity to HGF cells is crucial for improving treatment outcomes^6^.

Periodontal tissue damage is intricately linked to reactive oxygen species (ROS), predominantly produced by polymorphonuclear leukocytes^7^. Moreover, excessive free radicals resulting from oxidative stress or antioxidant deficiency contribute significantly to the development of periodontal disease^8^. When exposed to bacterial antigens, polymorphonuclear neutrophils (PMNs) are stimulated to generate free radicals through respiratory burst, rendering PMNs more active in patients with periodontal disease^9^. However, synthetic antioxidants like butylated hydroxytoluene (BHT) and butylated hydroxyanisole (BHA), commonly employed in efforts to restore periodontal health, can inflict DNA damage and exhibit carcinogenic effects on the liver^10^. Consequently, there is a growing interest in natural antioxidants as safer substitutes for synthetic ones in maintaining periodontal health^11^.

Basic nutrient requirements are met by plant-based foods, which also maintain the body in good health and provide defence from a variety of illnesses by strengthening immune function. Interest in black cumin (*Nigella sativa* L.), a highly prized nutritional herb with numerous health advantages, is developing among health-conscious people, scientists, and pharmaceutical firms^12^. *Nigella sativa*’s seeds, oil, and extract have demonstrated efficacy in treating ulcers, epilepsy, obese liver, types of arthritis, inflammatory diseases, cancers, and parasitic infections in humans^13,14^.

At a dosage of 10%, the black seed oil had bactericidal effects against streptococci and prevented their adhesion to tooth surfaces^15^. The literature revealed the use of *N. sativa* seed-based formulations, such as mouthwash^16^, nano-emulgel^17^, etc., in maintaining oral health. *N. sativa-*based toothpaste reduced swelling and fixed damaged bone matrix in rat periodontitis^18^. The potent anti-inflammatory and antioxidant properties of *N. sativa* (i.e., seed oil) are considered to be responsible for their beneficial effects on health^19,20^.

*Nigella sativa* seed essential oil (NSEO) holds promise in showcasing its potential to revolutionize oral health maintenance. Hitherto, different researchers have explored the potential of NSEO against different oral and periodontal pathogens^21–24^. However, to unlock its unprecedented benefits effectively, it is imperative to reveal its antioxidant and antimicrobial activity, specifically targeting periodontopathic microorganisms. This entails determining the oil's IC50 value through a series of tests, assessing its minimal inhibitory concentration against oral pathogens, and comprehensively understanding the underlying mechanisms of action. As such, ongoing research endeavours are centred on the task of isolating NSEO capable of combating oral pathogens while simultaneously preserving the delicate balance of the oral microenvironment and ensuring superior biocompatibility. Though literature exists regarding the beneficial properties of *N. sativa* oil, there is paucity in comprehensive data concerning its antimicrobial potential against periodontal pathogens. Also, there is very limited literature in dentistry regarding biological activities such as heat induced hemolysis and HRBC stabilizing assays conducted in that could be utilized for combatting periodontal inflammation.

Given the glaring gap in research surrounding NSEO's impact on oral health, our study ventures into uncharted territory to systematically explore its effects on key oral parameters. Specifically, we aim to the assessment of its antibacterial efficacy against a spectrum of periodontal pathogens, including *A. actinomycetemcomitans*, *P. gingivalis*, *T. forsythia*, and *P. intermedia*, utilizing techniques such as the agar well diffusion method. Moreover, our approach involves evaluating its anti-inflammatory responses and antioxidant activity, setting a new standard for comprehensive analysis. Additionally, our research extends to conducting cytocompatibility tests with primary human gingival fibroblast cell lines, marking a significant leap forward in validating safety *in-vitro*. Through this trailblazing endeavour, we strive to unravel the untapped potential of NSEO in dental care, offering insights that have the power to redefine approaches to oral health maintenance and treatment.

## Materials and methods

### Materials and chemicals used

*N. sativa* seeds were purchased from FRLHT institute, Bangalore. NaOH, HCl, NaNO2, ethyl alcohol, petroleum ether, H2SO4, trichloroacetic acid, ascorbic acid, ferric chloride, nitro-blue tetrazolium (NBT), nicotinamide adenine dinucleotide, and phenazinemethosulphate were all procured from Quligen Pvt. Ltd. Folin-Ciocalteu reagent, gallic acid, quercetin, DPPH, potassium ferricyanide [K3Fe(CN)6], 2, 2′-azino-bis (3-ethylbenzothiazoline-6-sulfonic acid) diammonium salt (ABTS), potassium persulfate (K2S2O8), nutrient agar, streptomycin, resazurin, DMEM medium, and MTT reagent were obtained from Himedia Pvt. Ltd. Additionally, Na_2_CO_3_ was sourced from Loba Chemicals.

### Authentication of raw sample

This study was conducted according to the guidelines of the Declaration of Helsinki, and approved by the Institutional Review Board (or Ethics Committee) of Universiti Sains Malaysia (JEPeM-USM), with protocol code USM/JEPeM/KK/23030217. *N. sativa* seeds were obtained from the reputed institute, Foundation for Revitalization of Local Health Traditions, Bangalore, India (FRLHT No-5693). The *N. sativa* plant and seeds were identified taxonomically for their authenticity by a senior botanist, at Infinite Biotech Institute of Research, Sangli, India. The seeds were then thoroughly washed with distilled water, shade dried, and identified by the herbarium and a voucher (IBR#5022) specimen of plant species was deposited for further reference.

### Extraction of NSEO

Freshly collected *Nigella sativa* seeds (2.2 kg) underwent initial rinsing three times with 3 L of distilled water to remove debris. Subsequently, the seed coat was crushed using a motor and pestle to facilitate the extraction of essential oils via hydrodistillation. The hydrodistillation process was conducted using a Clevenger apparatus under optimized conditions, maintaining a temperature of 55 °C. For hydrodistillation, a mixture of 100 g of ground seeds and 500 mL of distilled water was prepared and subjected to hydrodistillation for duration of 3 h. The essential oils released during the hydrodistillation process were collected. To remove any residual moisture, anhydrous sodium sulfate was employed to dry the obtained *N. sativa* essential oil (NSEO). The resulting oil exhibited a characteristic dark yellow color and a potent aroma indicative of its purity and concentration. Finally, NSEO was stored in opaque containers at a temperature of 4 °C until further use, ensuring preservation of its quality and efficacy^25,26^.

### Compositional characterization of NSEO

#### Phytochemical investigation

Qualitative assays were used to corroborate the presence of secondary metabolites in the extracted NSEO. Tests for flavonoids, glycosides, carbohydrates, saponins, alkaloids, steroids, phenols, terpenoids, starch, proteins, etc*.*, were performed^27^. To detect glycosides, Keller Killani, Raymond's, and Legal's tests were performed. Alkaloids were detected using Wagner’s test, Mayer’s test, as well as Hager’s reagent. Multiple tests like zinc hydrochloric acid-reduction, ferric chloride, Shinoda, lead acetate solution, along with, alkaline reagent were undertaken to identify flavonoids. The presence of steroids was corroborated using chloroform and Salkowski's tests. A ferric chloride test was employed to detect phenols, while terpenoids were detected using Salkowaski and Liebermann-Burchard test. Foam, hemolysis, and Raymond's tests were used to detect saponins. The presence of carbohydrates was confirmed using different tests, namely Molisch's, Barfoed's, and Benedict's tests. Proteins were identified by Biuret, xanthoproteic, and Millon's tests. Tannins were detected through NaOH test and Gelatin test. HCl test was used to identify anthocyanins^28^.

#### Gas chromatography-tandem spectrophotometry-MS (GC–MS/MS) analysis

A Shimadzu 17A GC coupled with a QP5050A (quadruple) Mass Spectrometer (Shimadzu, Japan) was utilized for the analysis. It was equipped with EI and a fused silica column DB-5 (30 m × 0.25 mm) with a film thickness of 0.25 µm. The oven temperature was initially set at 50 °C for 5 min and then programmed to increase from 50 to 280 °C for 40 min. A helium flow rate of 2 ml/minute was maintained; with a split ratio of 1:30 for sample injection of 1 µl. MS-analysis was performed using EI technique with an ionization voltage of 70 eV. The constituents of NSEO were identified by matching their MS and retention index data with those of standard ethnic spectra and by comparing their fragmentation pattern in Mass Spectra with those of WILEY 139.LIB and NIST 12.LIB. Retention indices were calculated using Kovats's procedure.

#### Total-content analysis

##### Phenols

The overall phenolic amount of NSEO was estimated using the Folin-Ciocalteu approach. In short, 10% (w/v) Folin-Ciocalteu reagent (2.5 mL) was combined with 1 mL of NSEO (300–900 µg/mL). Post-five minutes, Na_2_CO_3_ (2.0 mL; 75% conc.) was introduced to the blend, following the incubation for 10 min at 50 °C with periodic stirring. After cooling, the absorbance at 765 nm was estimated by an UV Spectrophotometer (Shimazu, UV-1800, Tokyo, Japan). Based on a gallic acid standard curve (200–1000 µg/mL), the results were represented as µg/g of GAE in milligrams per gram (µg GAE/g) of dry material^29^.

##### Flavonoids

To estimate the total flavonoid content in NSEO, a colorimetric method was employed. Initially, 1 mL of NSEO was combined with 0.3 mL of NaNO_2_ solution (5%), followed by dilution with 4 mL of distilled water. Subsequently, 2.4 mL of distilled water, 2 mL of 1 M NaOH, and 0.3 mL of 10% AlCl_3_ H_2_O solution were sequentially added to the mixture. After thorough mixing, the absorbance of the resulting solution was measured at 510 nm using a spectrophotometer. To generate standard curves, solutions of quercetin ranging from 200 to 1000 µg/mL were prepared and subjected to the same procedure. The absorbance values obtained from the standard solutions were plotted against their respective concentrations to construct the standard curves. Finally, the total flavonoid concentration in NSEO was determined by comparing its absorbance value with the standard curves, and the results were expressed in quercetin equivalents (QE) per gram of extract. This method provides an effective means of quantifying the flavonoid content in NSEO, facilitating its characterization and potential applications in various fields^30^.

##### Terpenoids

To determine the total terpenoid content in NSEO, we employed the method described by Ferguson (1956). Initially, 2 mL of NSEO at various concentrations was placed in a conical flask and allowed to soak in ethyl alcohol for duration of one day. This step ensured the dissolution of terpenoids present in the essential oil into the alcohol solvent. Following the soaking period, the mixture underwent filtration to remove any insoluble particles or impurities. The filtrate obtained was then subjected to extraction with petroleum ether. Petroleum ether, being a non-polar solvent, efficiently extracted the terpenoid compounds from the alcoholic solution. After extraction, the petroleum ether layer, containing the terpenoids, was separated and collected. This ether extract was considered as the measure of the total terpenoid content present in the NSEO sample. Total terpenoid content was measured using following Eq. (1),

1 $${\text{Total terpenoid content }} = \, ({\text{Final weight of the sample }} - {\text{ Initial weight of the extract/Weight of the Sample }}) \times \, 100$$

##### Steroids

To estimate the total steroid content in NSEO, we utilized a colorimetric method. Initially, 1 mL of the test extract containing steroids was transferred into a 10 mL volumetric flask. To this, 2 mL of 4N H_2_SO_4_ and 2 mL of 0.5% w/v FeCl_3_ solution were added, followed by the addition of 0.5 mL of 0.5% w/v K_4_[Fe(CN)_6_] solution. The resulting mixture was then subjected to heating in a water-bath maintained at a temperature of 70 ± 20 °C for duration of 30 min, with intermittent shaking to ensure uniform mixing. Following heating, the volume of the mixture was adjusted to the mark with distilled water in the volumetric flask. Subsequently, the absorbance of the solution was measured at a wavelength of 780 nm against a reagent blank. This absorbance reading provided a quantitative measure of the total steroid content present in the NSEO sample.

### Analytical characterization of NSEO

#### UV visible spectroscopy

A UV–Vis spectrometer (V-570, Jasco, Japan) with the 200–800 nm scanning range was used to record the UV–Vis absorption spectra of NSEO. The extracted NSEO was scanned for spectral run using UV 1800 Model of Shimadzu from 800 to 200 nm scanning range for 120 s run time. For this purpose, 2 mL of NSEO in the liquid form was kept in the UV–Vis cuvette.

#### FT-IR spectroscopy

To acquire the FTIR spectrum of NSEO, we employed a Bruker ALPHA II FTIR spectrophotometer, capable of scanning wavelengths from 4000 to 400 cm^−1^ with a high resolution of 2 cm^−1^. Initially, KBr pellets were prepared as the sample holder for analysis. Next, a small drop of NSEO was carefully applied onto the surface of a KBr pellet. It was crucial to ensure that only a minimal amount of NSEO was added to prevent excessive sample thickness, which could distort the FTIR signal. After application, any excess NSEO on the surface of the KBr pellet was meticulously removed using a capillary tube to achieve a uniform and thin layer of the sample. Subsequently, the prepared pellets were dried to remove any residual moisture or solvent, ensuring optimal conditions for FTIR analysis. Finally, FTIR spectra were recorded for the dried NSEO-loaded KBr pellets, allowing for the identification of characteristic absorption bands corresponding to functional groups present in the NSEO sample.

#### Zeta potential

The zeta potential of NSEO was assessed using a Horiba Scientific SZ-100 instrument. Following fresh extraction, the sample was appropriately diluted with Dimethoxysulfoxide (DMSO). Electrophoretic mobility measurements were conducted at a temperature of 25 °C and a dispersant dielectric constant of 78.5. The obtained electrophoretic mobility values were then input into Horiba Version 2.40 software to calculate the zeta potential.

### Antioxidant activity

#### Antioxidant activity by DPPH radical scavenging assay

The assessment of antioxidant activity in the NSEO was conducted through a DPPH (2,2-diphenyl-1-picrylhydrazyl) free radical scavenging assay, a widely used method to evaluate the antioxidant capacity of natural products. To begin the experiment, various concentrations of NSEO ranging from 200 to 1000 µg/mL were prepared, along with standard solutions of ascorbic acid and positive controls of Chlorhexidine gluconate (CHX 0.2%). Next, 1.5 mL of a 0.1% methanolic DPPH solution was added to each test tube containing the NSEO samples and controls. DPPH is a stable free radical with a deep purple color, which fades upon reaction with an antioxidant compound. The test tubes were then incubated in darkness for 30 min. This incubation period allows for sufficient time for the NSEO compounds to react with the DPPH radicals and neutralize them, leading to a discoloration of the solution from purple to yellow or red. Following the incubation period, the degree of discoloration in each test tube was visually assessed. The extent of discoloration correlates with the antioxidant activity of NSEO, with greater discoloration indicating higher scavenging activity against DPPH radicals. To quantify the antioxidant activity, the absorbance of each sample was measured at 510 nm using a colorimeter, such as the Labtronics LT-114. By comparing the absorbance values of NSEO samples with those of the standard solutions and positive controls, the antioxidant potential of NSEO was be determined. Radical scavenging activity was calculated by the following Eq. (2),

2 $${\text{DPPH radical scavenging activity}}\left( \% \right) = [({\text{Absorbance of control}} - {\text{Absorbance of test sample}})/({\text{Absorbance of control}})] \times 100$$

#### Ferric ion reducing capacity assay

The ferric reducing antioxidant capacity (FRAC) of samples was evaluated by the method of Oyaizu (Oyaizu, 1986). The Fe^2+^ can be monitored by measuring the formation of Perl’s Prussian blue at 700 nm. 0.25 mL NSEO, ascorbic acid and positive control CHX 0.2% at different concentrations (200, 400, 600, 800, 1000 μg/mL), 0.625 mL of potassium buffer (0.2 M, pH-6.9) and 0.625 mL of 1% potassium ferricyanide [K_3_Fe (CN)_6_] solution was added into the test tubes. The reaction mixtures were incubated for 20 min at 50 °C to complete the reaction. Then 0.625 mL of 10% trichloro acetic acid (TCA) solution was added to the test tubes. The total mixture was centrifuged at 3000 rpm for 10 min, after which 1.8 mL supernatant was withdrawn from the test tubes and mixed with 1.8 mL of distilled water and 0.36 mL of 0.1% ferric chloride (FeCl_3_) solution. The absorbance of the solution was measured at 700 nm using a spectrophotometer (Labtronics LT-116) against blank. The percentage of FRAC by the following Eq.(3),

3 $${\text{FRAC}} = \left( {{\text{OD of control}} - {\text{OD of Test/OD of Control}}} \right) \times 100$$

#### ABTS + radical scavenging assay

The assessment of antioxidant activity in NSEO involved an ABTS^+^ radical cation scavenging assay with slight modifications. Initially, the ABTS + radical cation was generated by combining 7 mM 2,2'-azino-bis(3-ethylbenzothiazoline-6-sulfonic acid) diammonium salt (ABTS) with 2.45 mM potassium persulfate (K_2_S_2_O_8_), followed by incubation at room temperature in the absence of light. To evaluate the ABTS radical scavenging activity, 3 mL of the prepared ABTS^+^ solution was mixed thoroughly with 0.2 mL of various concentrations (ranging from 200 to 1000 μg/mL) of NSEO, as well as standard solutions of ascorbic acid and a positive control of CHX 0.2%. The reaction mixture was then allowed to stand at room temperature for 6 min, facilitating the interaction between NSEO compounds and the ABTS^+^ radicals. This incubation period ensures sufficient time for the antioxidant components in NSEO to neutralize the ABTS radicals. Subsequently, the absorbance of the reaction mixture was measured spectrophotometrically at an appropriate wavelength. The decrease in absorbance indicates the scavenging ability of NSEO against ABTS radicals, with higher absorbance reductions corresponding to greater antioxidant activity. By comparing the scavenging activity of NSEO with that of the standard solutions and positive control, the antioxidant potential of NSEO can be determined. The percentage inhibition was calculated by the following Eq. (4),

4 $$\% {\text{Inhibition }} = \, ({\text{OD of control }} - {\text{ OD of sample/OD of control}}) \, \times \, 100$$

#### Super oxide dismutase (SOD) enzymatic assay

The superoxide anion scavenging activity of NSEO was assessed following a modified version of the method described by Robak and Gryglewski (1988). All solutions were prepared using 100 mM phosphate buffer at pH 7.4 to maintain consistent conditions throughout the assay. In a reaction mixture, 100 μL of nitro-blue tetrazolium (NBT, 156 μM), 50 μL of reduced nicotinamide adenine dinucleotide (NADH, 468 μM), and 100 μL of NSEO, ascorbic acid, and CHX 0.2% solutions at various concentrations (ranging from 200 to 1000 μg/mL) were combined. The reaction was initiated by adding 10 μL of phenazinemethosulphate (PMS, 60 μM) to the mixture. Subsequently, the reaction mixture was incubated at 25 °C for 5 min to allow for the scavenging of superoxide anions by the test compounds. After the incubation period, the absorbance of the reaction mixture was measured spectrophotometrically at 560 nm. The decrease in absorbance indicates the scavenging activity of NSEO against superoxide anions, with higher reductions in absorbance corresponding to greater scavenging efficacy. By comparing the scavenging activity of NSEO with that of ascorbic acid and the positive control CHX 0.2% across different concentrations, the ability of NSEO to neutralize superoxide anions was evaluated. The percentage inhibition was calculated by using Eq. (5),

5 $${\text{Percentage inhibition }} = \, ({\text{Control}} - {\text{test/absorbance of control}}) \, \times \, 100$$

### Anti-inflammatory activity

#### Protein denaturation activity

To assess the anti-inflammatory potential of NSEO, a solution containing varying concentrations of NSEO (100 µL), phosphate-buffered saline (PBS) adjusted to pH 6.4 (5.6 mL), and fresh hen's egg albumin (0.4 mL) was prepared, yielding a final volume of 10 mL. Following thorough mixing, the solution was allowed to incubate for 15 min at 37 °C to facilitate interaction between the components. Subsequently, the mixture was subjected to a 5-min heat treatment at 70 °C to induce denaturation of the protein. After cooling to room temperature, the turbidity of the solution was measured at 660 nm using an Optima SP-3000 UV/VIS spectrometer (Tokyo, Japan). Diclofenac sodium served as the standard reference, while PBS was used as a control to establish baseline turbidity levels. By comparing the turbidity of the NSEO-treated samples with that of the control and standard, the ability of NSEO to inhibit protein denaturation was evaluated. The percentage of protein denaturation inhibition was determined using Eq. (6):

6 $$\% {\text{ inhibition of denaturation}} = 100 \times \left( {1 - T/C} \right)$$

where C is the control sample's absorption and T is the test sample's absorption^31^.

#### Human red blood cell (HRBC) stabilizing assay

To perform the HRBC membrane stabilization experiment, 5 mL of blood was drawn from a healthy donor who had abstained from nonsteroidal anti-inflammatory drugs (NSAIDs) for at least 15 days. This blood sample was then mixed with an Alsever solution, consisting of dextrose (20.5 g), citric acid (0.55 g), sodium citrate (8 g), and sodium chloride (4.2 g) dissolved in distilled water (1000 mL). After mixing, the blood-Alsever solution mixture was centrifuged at 3000×*g* for 15 min to separate the blood cells. The resulting cell pellet was washed with an isosaline solution to remove any residual components. Next, an assay mixture was prepared, comprising 0.15 M PBS (1 mL; pH 7.4), HRBC suspension (500 µL), 0.36% hyposaline solution (2 mL), and NSEO (500 µL). This assay mixture was then incubated in a BOD incubator at 37 °C for 30 min to allow for stabilization. Following incubation, the suspension underwent centrifugation at 3000×*g* for 20 min. Aspirin and deionized water were included as positive and negative controls, respectively. The amount of hemoglobin released into the supernatant was measured using a UV–Visible spectrophotometer by recording the absorbance at 560 nm^32^. The % of HRBC membrane stabilization was measured using Eq. (7):

7 $${\text{Inhibition}}( \% ) = 100 - \left( {OD_{s} /OD_{c} } \right) \times 100$$

where OD_c_ is the control’ absorbance and OD_s_ is the sample’s optical density.

#### Heat-induced hemolysis

In brief, PBS (2.95 mL; pH 7.4) was mixed with varying concentrations of NSEO (0.05 mL) and blood cell suspension (0.05 mL) ranging from 200 to 1000 µL/mL. The resulting mixture was then incubated at 54 °C for 20 min. Following 5-min incubation at 5000 rpm, the absorbance of the supernatant was measured at 540 nm using a UV/Vis spectrometer. Aspirin (200–1000 µg/mL) and phosphate buffer solution served as the positive and negative controls for the tests, respectively. Equation (8) was used to measure the degree of hemolysis^31^,

8 $$\% {\text{inhibition of hemolysis}} = 100 \times \left( {1 - T/C} \right)$$

### Antimicrobial activity

#### Antibacterial activity against oral pathogens

The antibacterial and antifungal activities of NSEO were evaluated using the agar well diffusion method. For the antifungal assay, fungal strains including *Aspergillus niger* and *Candida albicans* were cultured on Sabouraud Dextrose agar plates, which provide optimal growth conditions for fungi. Meanwhile, for the antibacterial assay, bacterial strains such as *L. acidophilus*, *E. coli*, *S. aureus*, and *P. aeruginosa* were inoculated onto nutrient agar plates, which support the growth of a wide range of bacteria. The inoculum concentration was standardized to 1.5 × 10^8^ cells/mL to ensure consistency in the experimental setup. Once the agar plates were prepared with the respective microbial cultures, they were allowed to dry to ensure proper absorption of the test samples. Cork borer wells were then created on the agar plates using a sterile cork borer of appropriate diameter. Solutions of the test compounds, including NSEO at a concentration of 100 µL/mL, were prepared in dimethyl sulfoxide (DMSO), a commonly used solvent for dissolving hydrophobic compounds. Subsequently, 100 µL of each test sample solution was carefully introduced into the wells created on the agar plates. Negative controls, consisting of DMSO alone, were included to account for any potential solvent effects on microbial growth. Positive controls were also included to validate the assay, with streptomycin (1 µg/mL) used as the positive control for antibacterial activity and miconazole (1 g/mL) for antifungal activity. Following sample introduction, the agar plates were incubated at 37 °C for 24 h to allow for microbial growth and diffusion of the test samples into the surrounding agar medium. After the incubation period, the plates were examined for the presence of zones of inhibition around the wells where the test samples were introduced. The diameter of the zones of inhibition was measured using a calibrated ruler or digital caliper to assess the extent of microbial growth inhibition. Additionally, IC50 values, representing the concentration of the test sample required to inhibit microbial growth by 50%, were calculated using appropriate statistical methods^32^.

#### Antibacterial activity against periodontal pathogens

Using the agar well diffusion method, the antibacterial activity of extracted NSEO was tested against pathogenic periodontal bacteria (*A. actinomycetemcomitans, P. intermedia, T, forsythia* and *P. gingivalis*). Briefly, bacterial strains (1.5 × 10^8^ cells/mL) were seeded into nutrient agar plate. After drying the plates, cork borer wells were formed. Solutions of the compounds (100 µL/mL) were prepared in DMSO, and 100 µL of samples were introduced to wells. For antibacterial activity, DMSO as a negative control and streptomycin (1 µg/mL) was employed as a positive control. To cultivate these organisms, anerobic conditions were maintained to ensure their growth and viability, differing significantly from aerobic cultivation. Firstly, anaerobic environment was achieved using anaerobic incubators that maintained an atmosphere typically composed of 85% nitrogen, 10% hydrogen, and 5% CO_2_. The culture media used for anaerobes were pre-reduced by adding reducing agents like thioglycolate or cysteine and then sealed to exclude oxygen before sterilization. The incubation temperature for anaerobes was maintained at 37 °C, and high humidity within anaerobic chambers was also maintained to prevent desiccation. The pH of the media was typically maintained between 7.2 and 7.6, and anaerobic indicators like resazurin or methylene blue were used to ensure the absence of oxygen. This was followed by calculating the zone of inhibition in mm^32^.

#### Minimum inhibitory concentration (MIC)

The MIC of NSEO was evaluated by resazurin assay method^33^. The assay was prepared by dissolving 270 mg of resazurin in 40 mL of sterile distilled water. A vortex mixer was used to make sure that the solution was homogenous and dissolved. 96-well plate under aseptic conditions was used to carry out the studies. A sterile 96-well plate was labeled. 100 μL volume of different concentrations (7.8, 15.6, 31.2, 62.5, 125, 250, 500, 1000 µg/ mL) of NSEO, standard and positive control (CHX 0.2%) was pipetted first into the well of the plate. Then, 50 μL of nutrient broth was added to all different wells and subsequently diluted. To each well, 10 μL of resazurin indicator solution was added. After this, 10 μL of fungal or bacterial suspension was added to every well. Using a cling film, every plate was covered loosely to avoid the dehydration of microbes. Then, the plate was incubated for 18–24 h at 37 °C and colour change was studied visually. Any colour variations from blue to pink or colourless were denoted as positive and the absence of colour change was indicated as negative. The lowest concentration of the sample at which the colour change was observed was considered as the minimum inhibitory concentration (MIC) value. And the absorbance of the plate was measured at 600 nm by using ELISA reader. MIC was calculated using Eq. (9),

The percentage of Inhibition was calculated by the following formula:

9 $$\% {\text{ of Inhibition}} = \left( {{\text{Control}} - {\text{ Test/Control}}} \right) \times {1}00$$

#### Minimum bactericidal concentration (MBC)

After determining the minimum inhibitory concentration (MIC) of the NSEO, 50 μL aliquots from wells showing no visible bacterial growth were transferred onto Brain Heart Infusion (BHI) agar plates. These plates were then incubated at 37 °C for 24 h to allow any surviving bacteria to form visible colonies. The minimum bactericidal concentration (MBC) endpoint, defined as the lowest concentration of the antimicrobial agent at which 99.9% of the bacterial population is killed, was determined by comparing the bacterial growth on pre- and post-incubation agar plates to assess the presence or absence of viable bacteria^34^.

### Cytocompatibility of NSEO over human gingival fibroblast cell lines

The HGF cell lines (hTERT Gingival Fibroblast CRL-4061TM) were cultured in a DMEM medium with 10% (v/v) FBS, Antibiotic – Antimycotic 100X solution 1% in 75 cm^2^ cell culture flasks kept at 37 °C in an incubator with 5% CO_2._ The culture was monitored under the microscope every two days, dissociated, and divided when the cell was near 100% confluent. Cells concentration was counted in the Thoma cell counting chamber.

Cells were incubated at a concentration of 1 × 10^4^ cells/mL in culture medium for 24 h at 37 °C and at 5% CO_2_. Cells were seeded at a concentration (100 μl) 10^4^ cells/well) in 100 μL culture medium and different concentrations of µg/mL of samples into microplates respectively (tissue culture grade, and 96 wells). Control wells were incubated with DMSO (0.2% in PBS) and cell lines. All samples were incubated in triplicate. To calculate the proportion of live cells after culture and the control cell survival, controls were kept. Cell cultures were incubated for 24 h at 37 °C and 5% CO_2_ in a CO_2_ incubator. After incubation, the medium was completely removed and 20 μL of MTT reagent (5 mg/mL in PBS) was added. After addition of MTT, cells were incubated for 4 h at 37 °C in a CO_2_ incubator. After removing the medium completely, 200 μL of DMSO (keep for 10 min) was added and incubated at 37 °C (wrapped with aluminium foil). Triplicate samples were analyzed by measuring the absorbance of each sample by a micro plate reader at a wavelength of 550 nm^35^. Percentage of viability was calculated by using following Eq. (10),

10 $$\% {\text{ of Viability}} = \left( {{\text{OD of Control}} - {\text{OD of Test/OD of Control}}} \right) \times {1}00$$

### Statistical analysis

Statistical difference between obtained results was estimated using students’ ‘t’ test and one-way ANOVA (analysis of variance) followed by Tukey HSD.

### Ethics approval and consent to participate

This study was conducted according to the guidelines of the Declaration of Helsinki and approved by the Institutional Review Board (or Ethics Committee) of Universiti Sains Malaysia (JEPeM-USM), with protocol code USM/JEPeM/KK/23030217. The informed consent was obtained from all subjects and/or their legal guardian(s).

## Results

### Extraction of NSEO

**Figure 1:**
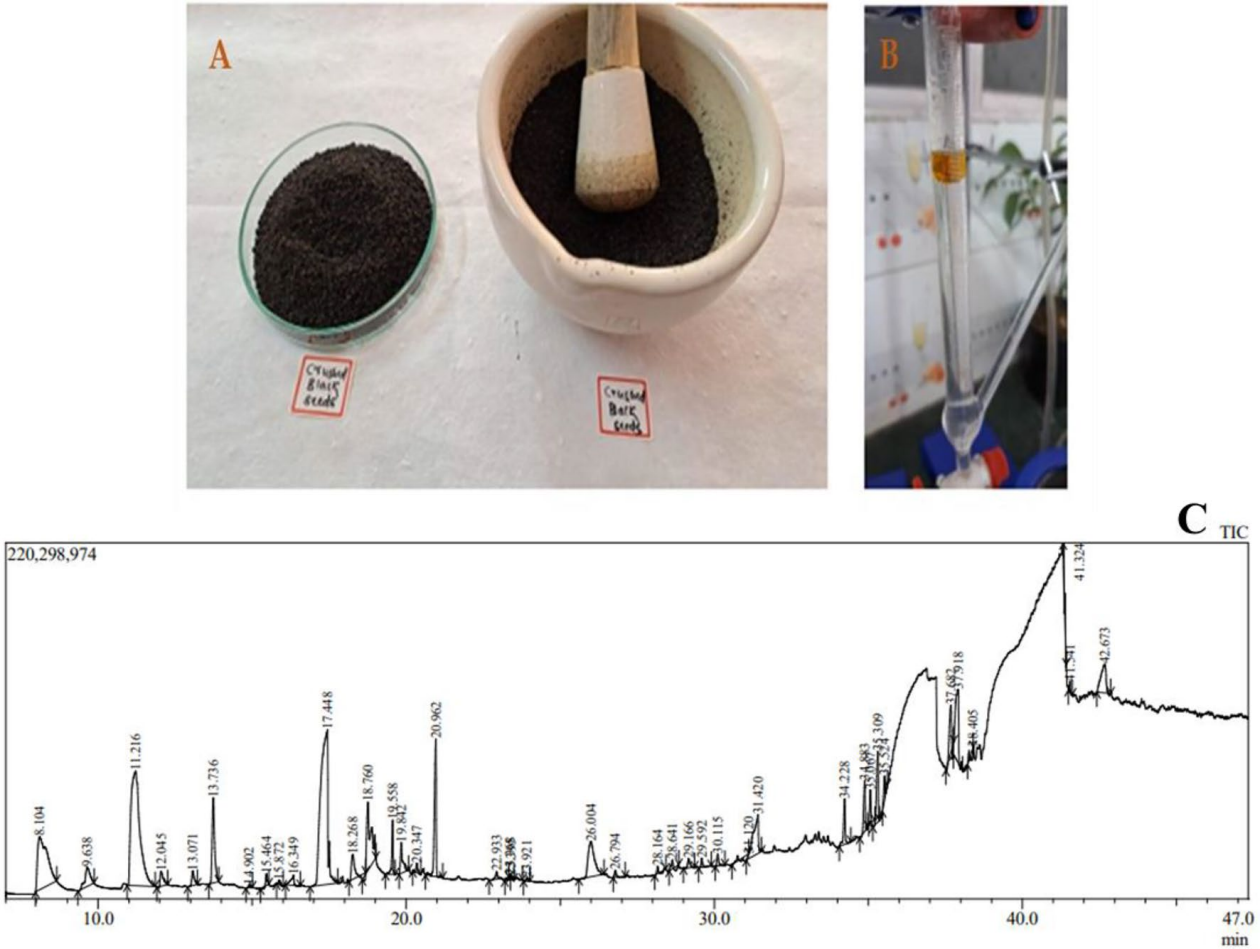
(**A**) *Nigella sativa* seeds crushed in mortar and pestle, (**B**) Extraction yield of NSEO by hydro-distillation technique, (**C**) GC–MS/MS Spectra of NSEO.

Pale yellow-colored NSEO was isolated using the hydro-distillation technique (Fig. 1A,B). 0.4 mL of oil was obtained per 100 gm of seeds. The resulting oil's color was observed to be light yellowish brown to brown.

### Phytochemical investigation

Investigation corroborated the availability of steroids, triterpenoids, alkaloids, phenols, proteins, flavonoids, and tannins.

### Composition characterization

#### Gas chromatography–mass spectrophotometry-MS (GC–MS MS)

The GC–MS/MS spectrum of NSEO is as shown in Fig. 1C. The GCMS/MS spectrum analysis of Nigella sativa essential oil (NSEO) revealed a diverse array of compounds present in the sample. Among these compounds, bicyclo[3.1.0]hex-2-ene,2-methyl-5-(1-methy and bicyclo[3.1.1]heptane,6,6-dimethyl-2-methyl, both identified as flavonoids, were detected at abundances of 9.83% and 1.87%, respectively. The terpenoid 1,3,8-p-Menthatriene was found to be the most abundant compound, constituting 19.74% of the oil. Other compounds identified include cis-4-methoxythujane (0.69%), longifolene (4.75%), and (1R,4R,5S)-1-Isopropyl-4-methoxy-4-methylb (4.11%), categorized as an oily alkylbenzene. Additionally, cyclohexen-1-ol,4-methyl-1-(1-methylethyl, a terpenoid, was detected at 0.33% abundance, along with 3,6-Octadienal,3,7-dimethyl- (0.11%) and (3E,5E)-2,6-Dimethylocta-3,5,7-trien-2-ol (0.13%), categorized as terpenoids and esters, respectively. Octanoic acid, identified as a fatty acid, was present at 0.60% abundance. Notably, 3,7,7-Trimethylbicyclo[4.1.0]hept-3-ene-2,5-d, classified as carene, constituted the highest abundance at 22.05%. Furthermore, 5-methyl-1,2,3,4-tetrahydropyrimidine-2,4-dione (5.37%), 2,4-Decadienal,(E,E) (1.04%), and 2-Tridecanone (0.22%) were also identified among the compounds detected. Tricyclo[5.4.0.0(2,8)]undec-9-ene,2,6,6,9-tetr and undecenal were present at 1.47% and 1.352% abundance, respectively. Finally, 1-Cyclohexene-1-carboxaldehyde, 2,6,6-trime, classified as thymoquinone, was detected at 0.32% abundance. These results provide insight into the chemical composition and relative abundances of bioactive compounds present in NSEO, contributing to its potential medicinal properties.

#### Total-content analysis

##### Phenols

The TPC of NSEO was estimated by Folin–Ciocalteu reagent. Moreover, results were obtained using gallic acid’s (200–1000 µg/mL) calibration curve (y = 0.0005x + 0.0184 R^2^ = 0.992) and are presented as GAE per gram dry extract weight (Table 1). The TPC was found to be 641.23 μg GAE/gm.

**Table 1:** Total phenolic and flavonoid contents of NSEO.

Sample	Conc. (µg/mL)	Total phenol content (μg GAE/g)	± SD	Total flavonoid content (μg QE/g)	± SD
NSEO	300	57.59	0.0047	37.25	0.816
	600	251.03	0.475	238.59	0.146
	900	641.23	0.857	442.25	0.047

##### Flavonoids

Flavonoid concentrations in NSEO were quantified using aluminium chloride in a colorimetric method as a framework for the quantitative analysis. Results were obtained from the calibration curve (y = 0.000x + 0.288; R^2^ = 0.973) of quercetin (0–100 µg/mL) and presented as QE per gram EO weight (Table 1). Total flavonoid content of NSEO at 900 µg/mL was found to be 443.25 (µg QE/g).

##### Terpenoids

**Figure 2:**
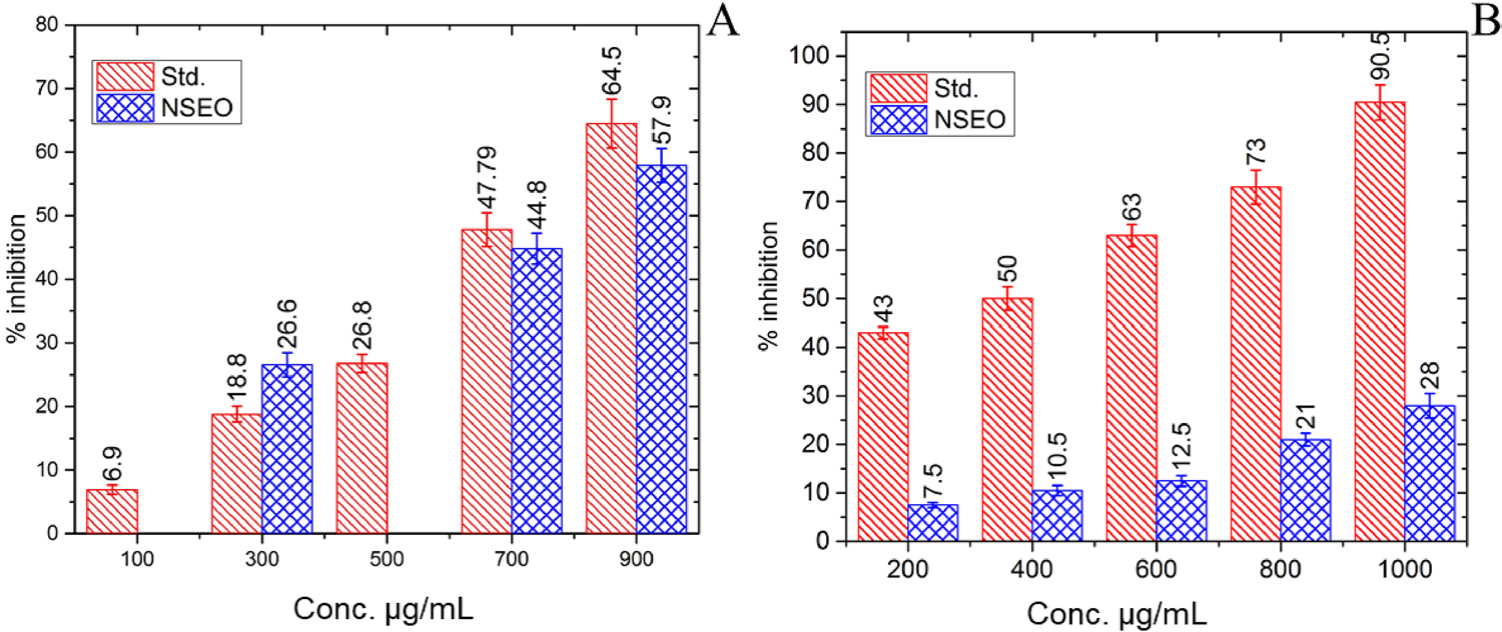
Graphical representation of (**A**) total terpenoid content and (**B**) total steroid content.

Terpenoids offer a wide range of health benefits, including anti-inflammatory, antioxidant, antimicrobial, neuroprotective, and anticancer effects, among others^36^. Total content of terpenoids in NSEO evaluated by colorimeter method. The assay shows that the NSEO contains 57.90% of total terpenoid content at 1000 µg/mL as compared to standard drug camphor. However, as the concentrations of NSEO increase from 300 to 900 µg/mL, NSEO shows the increased content of terpenoid as listed in Fig. 2A.

##### Steroids

Total content of steroids in NSEO was observed by colorimetric method. NSEO revealed small quantity of steroid was observed in various concentrations (200, 400, 600, 800, 1000 µg/mL) of NSEO as compared to Prednisone as summarized in Fig. 2B. However, the increased concentration of NSEO showed the increased percentage of steroid content in oil. NSEO revealed 28% of steroid content at its 1000 µg/mL concentration as compared to standard drug Prednisone.

### Analytical characterization of NSEO

#### UV Vis spectroscopy

**Figure 3:**
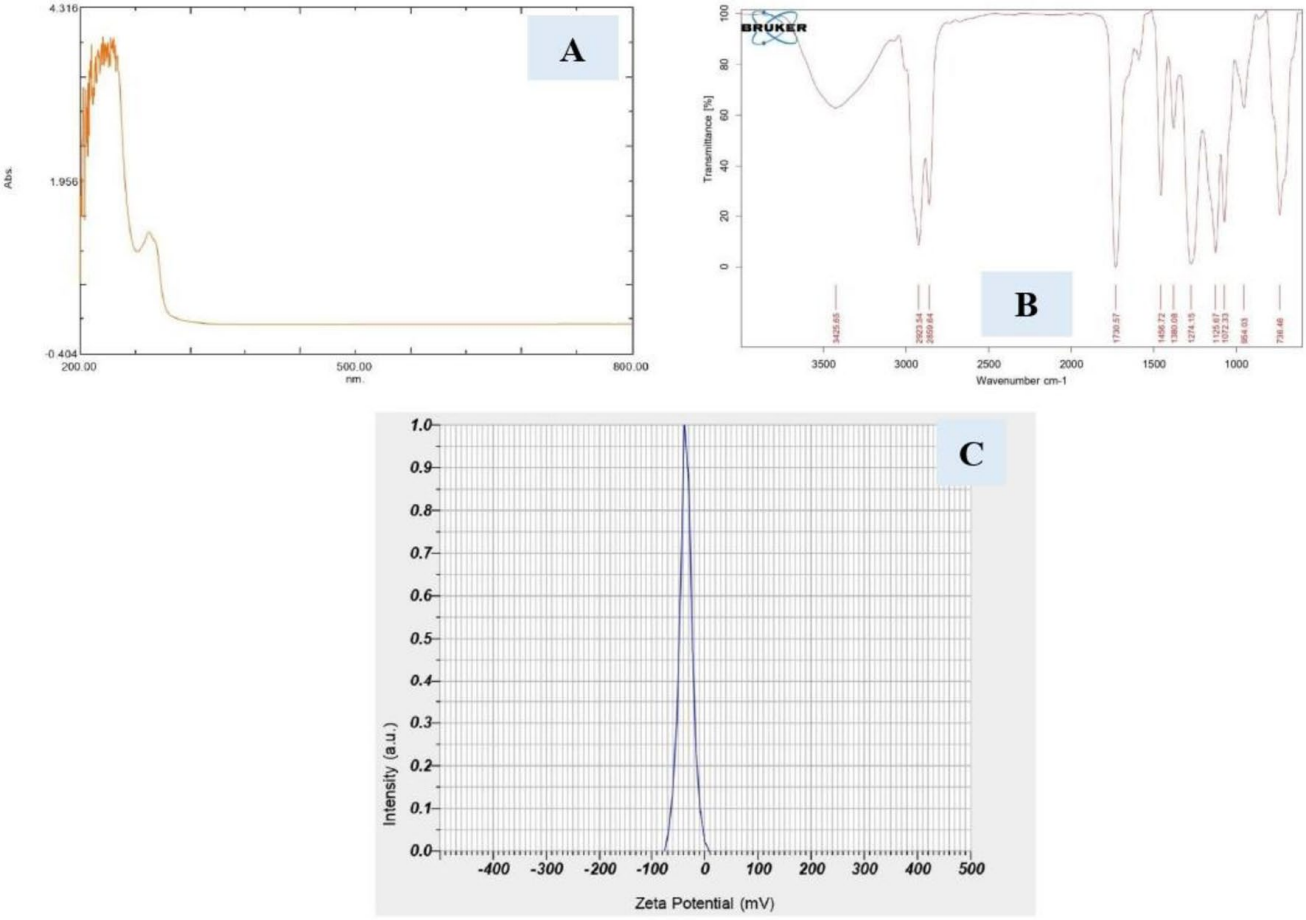
(**A**) UV Vis Spectrum of NSEO. (**B**) FT-IR Spectrum of NSEO. (**C**) Zeta potential of NSEO.

Two peaks were detected in the spectrum of NSEO as per the UV Spectrum as seen in Fig. 3A. The maximum absorbance was observed at 275 nm wavelength. Absorbance at wavelength below 400 nm indicates the presence of organic compounds without any major colour. At 10 µg/mL solution, high absorbance above 1.0 is observed. This means that the concentration of ester in oil is high as well as purity of the given oil is too high. The chemical constituents confirmed from the UV spectral graph was the esters. Pure essential oil containing ester without any colour usually shows absorbance at a wavelength within the range of 250–300 nm. Another peak was observed at 225 nm that may be due to secondary metabolites such as glycosides/flavonoids/alkaloids etc. In the UV spectrum, 275 nm was the highest wavelength reached when sample absorbed UV light and excited from ground stage to excited stage. Absorbance at around 254–257 also corroborated the presence of thymoquinone. Results are in line with previous reports^37,38^.

#### FT-IR spectroscopy

**Figure 4:**
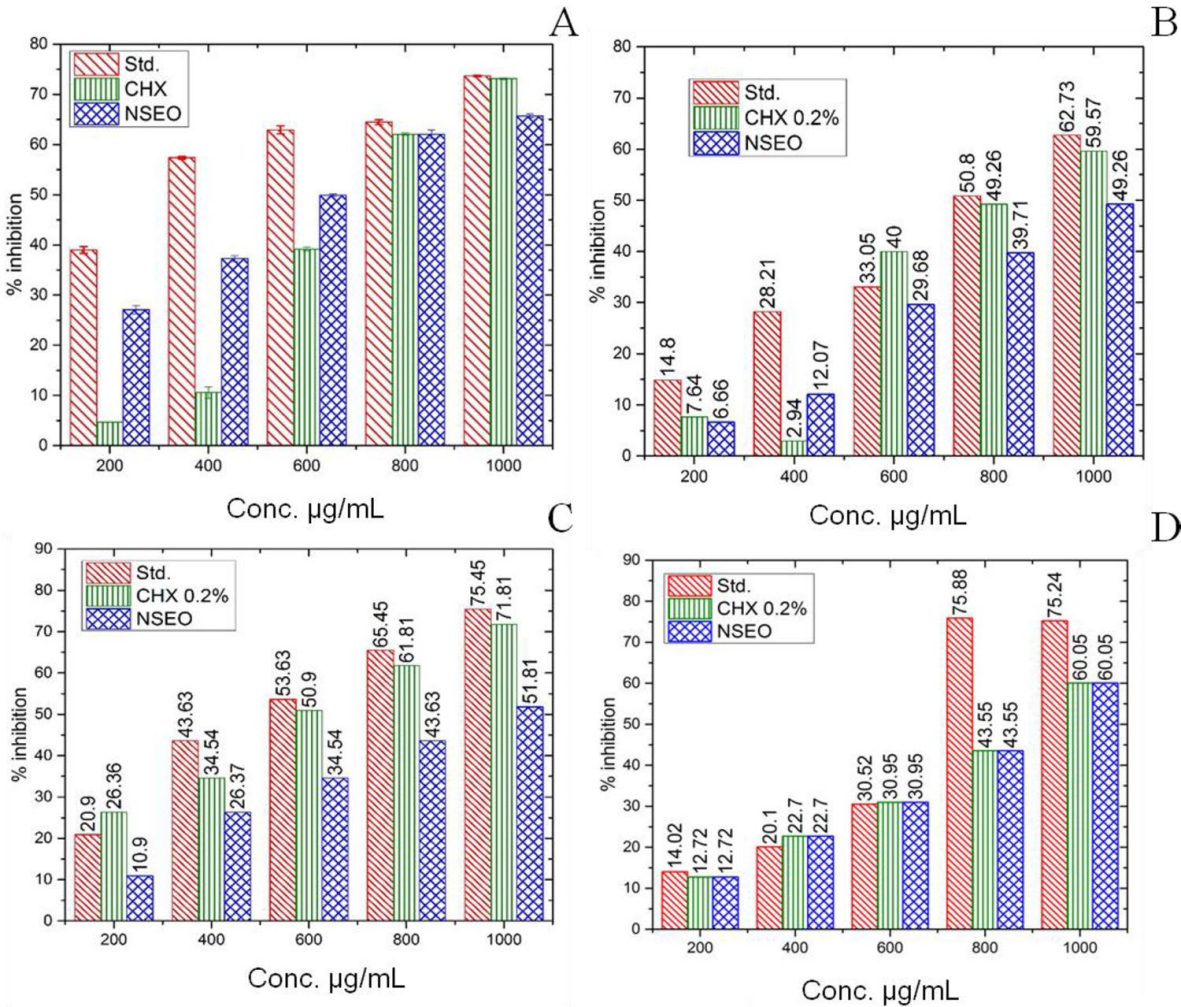
Graphical representation of percentage of inhibition against oxidants by (**A**) DPPH method and (**B**) FRAC method (**C**) ABTS method (**D**) SOD enzymatic assay.

The extracted NSEO was subjected to the FT-IR (Bruker Alpha II) to analyze its active functional groups by Attenuated Total Reflection Technique. The FT-IR spectra analysis revealed the presence of a diverse range of sharp peaks, including strong and weak signals, indicative of various functional groups such as C–H, –CH_2_, –CH_3_, C=O, C–O, and C=C. These findings strongly suggest the presence of major phenolic compounds including thymoquinone, dithymoquinone, thymohydroquinone, and thymol. Specifically, a weak absorption peak around 3010 cm^−1^ in the FT-IR spectrum (see Fig. 3B) corresponds to the C–H stretching of the vinyl group. Additionally, two intense bands observed at 2920 cm^−1^ and 2850 cm^−1^ are attributed to the C–H stretching of an aliphatic group, indicating the presence of methyl and isopropyl substituents. Furthermore, a significant strong band at 1730 cm^−1^ is identified as the C=O stretching of the ester group. Notably, an absorption band at ≈1630 cm^−1^ is observed, which is attributed to the C=O stretching of thymoquinone, suggesting a decrease in the resonance frequency effect of the carbonyl group (see Fig. 4). Moreover, the peaks at 1456 cm^−1^ and 1380 cm^−1^ are associated with C–H absorption scissoring and methyl rock motions, respectively. Additionally, weak peaks at 1125 cm^−1^ due to the C–O group and at 1072 cm^−1^ attributable to the =C–H bending group were observed. The presence of an ester group is indicated by the peak at 1274.15 cm^−1^ (Oil is an ester of fatty acid with glycerol). These observations align closely with findings reported in the existing literature^37,39^.

#### Zeta potential

Observed zeta potential value for NSEO was − 35.7 mV with electrophoretic mobility mean of − 0.000276 cm^2^/Vs at the temperature 24.9 °C, conductivity 0.138 ms/cm, electrode voltage 3.4 V and viscosity of dispersion medium 0.897 mpa/s. This spectrum falls under the category of moderately stable compound and nano zeta range. It is also confirmed that NSEO is negatively charged due to the presence of negative carboxylate ions in the oil. Zeta potential also confirms the presence of active compound due to presence of charge.

### Antioxidant activity

#### DPPH free radical assay

Five different concentrations of NSEO were tested ranging from 200 to 1000 µg/mL (Fig. 4A). NSEO shows the highest percentage of inhibition at 1000 µg/mL i.e., 65.80% as compared to the standard ascorbic acid (73.57%) and positive control CHX 0.2% (72.03%). NSEO inhibits the oxidants at lowest concentration, 200 µg/mL i.e., 27.97% and achieves approximately the same antioxidant power as compared to the standard drug ascorbic acid and positive control CHX 0.2%. IC_50_ values of standard, CHX 0.2% and NSEO were found to be 343.02, 680.45 and 615.2 µg/mL. Statistical significance (p < 0.5) between standard and CHX 0.2% was observed at concentrations 200, 400 and 600 µg/mL. Statistical significance (p < 0.00001) was observed between the results of ascorbic acid and CHX 0.2% compared to NSEO at all concentrations used, except at 800 µg/mL.

#### FRAC assay

NSEO showed good percentage of inhibition at 1000 µg/mL i.e., 49.26% as compared to the standard ascorbic acid (62.73%) and positive control CHX 0.2% (59.57%) (Fig. 4B). However, as the concentration increased, NSEO showed high percentage of inhibition. Statistical significance (p < 0.5) between standard and CHX 0.2% was observed at concentrations 600, 800 and 1000 µg/mL. Statistically significant results for standard compared to NSEO were observed at all used concentrations. However, when CHX 0.2% and NSEO were compared significance results were observed at 600, 800 and 1000 µg/mL between CHX 0.2% and NSEO.

#### ABTS + radical scavenging assay

The percentage of inhibition for NSEO was 51.81% at 1000 µg/mL as compared to positive control CHX 0.2% (41.81%) and standard (75.45%) at 1000 µg/mL (Fig. 4C). However, as concentrations of NSEO increased from 200 to 1000 µg/mL the percentage of inhibition increased, respectively. The highest radical scavenging activity for NSEO was observed at 1000 µg/mL. There was no statistical significance for absorbance values between standard and CHX 0.2% at all the used concentrations. However significant results were observed at concentrations 800 and 1000 µg/mL for standard in comparison with NSEO. Further statistical significance for CHX 0.2% in comparison with NSEO was observed at all concentrations except 400 µg/mL.

#### SOD enzymatic assay

The SOD activity assay relies on enzyme extract's ability to hinder NADH oxidation by superoxide radicals in a chemical system, measured spectrophotometrically by reduced NBT formation^40^. NSEO showed highest percentage of inhibition at 1000 µg/mL i.e., 60.06% as compared to the standard Curcumin (72.65%) and positive control CHX 0.2% (59.8%) (Fig. 4D). However, as the concentration of NSEO increased, the percentage of inhibition increased in a dose dependent manner. There was no statistical significance at all concentrations for standard and CHX 0.2%. However, statistical significance for standard and CHX 0.2% compared to NSEO was seen at 800 and 1000 µg/mL.

### Anti-inflammatory activities

#### Protein denaturation activity

**Figure 5:**
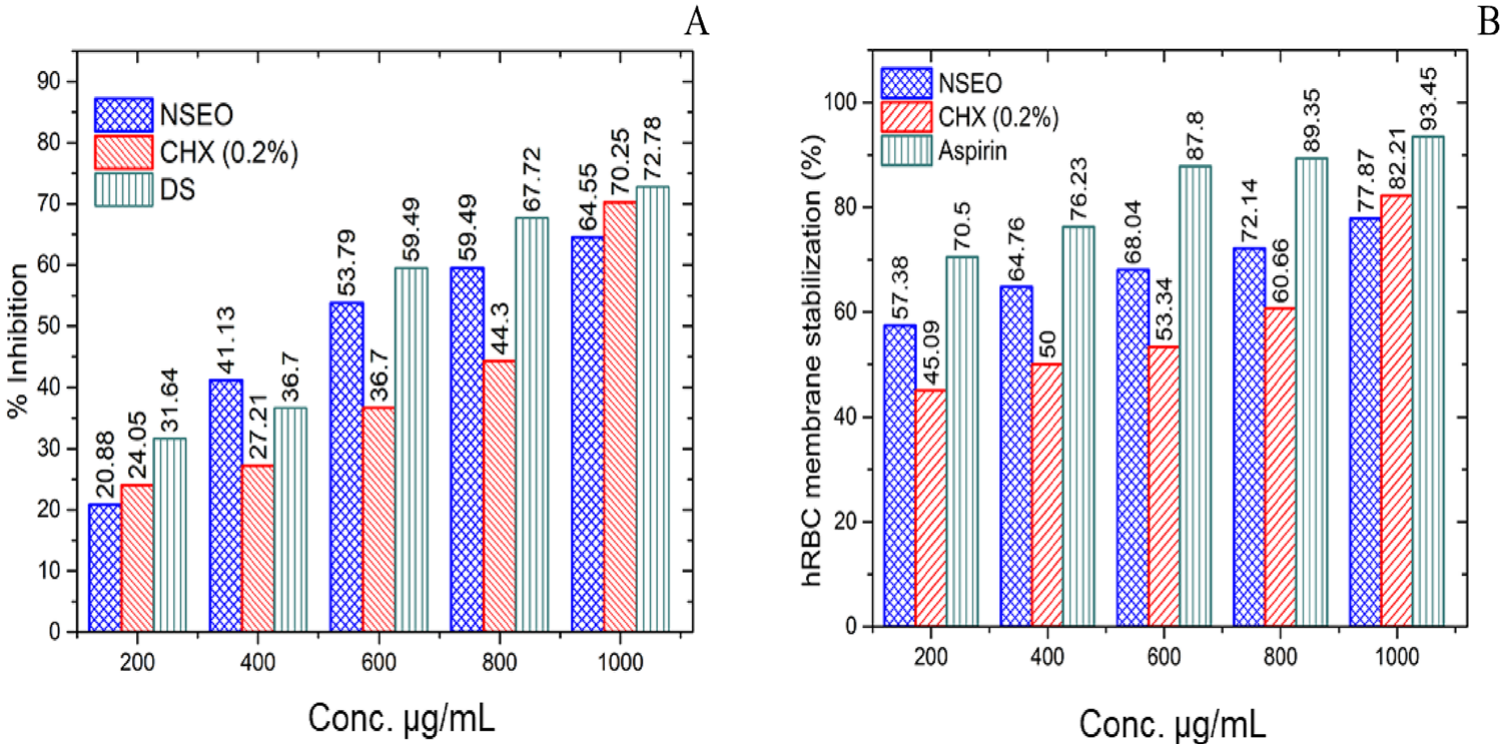
Graphical representation of (**A**) % inhibition of protein denaturation and (**B**) % stabilization of HRBC membrane by NSEO, CHX, and standard at different concentrations.

The current findings demonstrated a concentration-dependent inhibition of protein (albumin) denaturation by Diclofenac sodium, chlorhexidine (0.2%), and NSEO throughout the concentration range of 200–1000 μg/mL (Fig. 5A). The increased % inhibition in the NSEO, CHX and the standard concerning control indicates the protein stabilizing activity with increased dose. A statistical significance (p < 0.00001) was reported for the IC_50_ values of standard (490.23 µL/mL), CHX (900.25 µL/mL), and NSEO (540.36 µL/mL). Notably, NSEO exhibited a lesser IC50 value than CHX. Research findings indicate that the protein denaturation inhibition capacity of *Nigella sativa* seed cake was measured at 150.39 ± 2.61 μg/mL, showcasing its potential in preserving protein structure^41^. Notably, the NSEO demonstrated a remarkable inhibition rate, reaching a peak of 82.966 ± 3.704% at a concentration of 500 µg/mL^42^.

#### HRBC stabilizing assay

Given that the HRBC membrane mimics the lysosomal membrane and may be inferred to the stabilization of the lysosomal membrane, it can be used to study anti-inflammatory efficacy *in-vitro*^43^. HRBC membrane stabilization refers to the ability of certain substances or conditions to safeguard the cell membrane against various destabilizing factors, such as oxidative stress or exposure to certain chemicals. Standard (aspirin), NSEO, and CHX exhibited concentration-dependent stabilization of the HRBC membrane.

At concentrations between 200 and 1000 µL/mL, NSEO may protect lysis of human erythrocytes stimulated using a hypotonic solution. 1000 µg/mL of NSEO and CHX 0.2% inhibited 77.87% and 82.21% of RBC haemolysis, whereas Aspirin inhibited 93.45% (Fig. 5B). The results revealed that CHX 0.2% and sample NSEO can inhibit HRBC hemolysis remarkably and in a dose-dependent manner.

#### Heat-induced hemolysis

**Figure 6:**
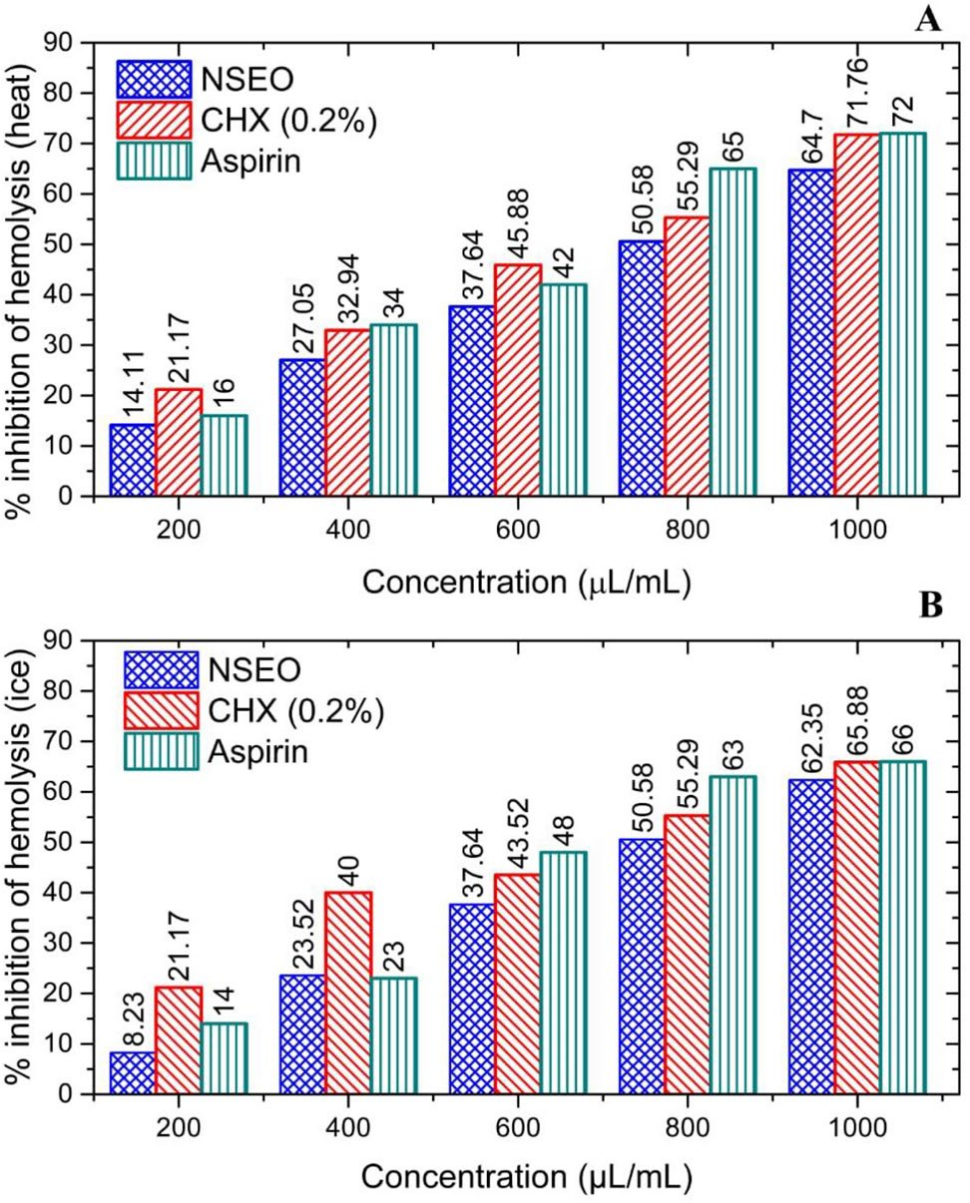
Percent inhibition of (**A**) heat and (**B**) ice-induced hemolysis by NSEO, CHX, and aspirin.

Heat-induced hemolysis can lead to the release of pro-inflammatory mediators such as cell-free hemoglobin, heme, and other cellular debris. These substances can activate inflammatory pathways and stimulate immune responses. Therefore, inhibition of heat-induced hemolysis could offer protection from inflammation. NSEO protects RBC from heat-induced hemolysis, and its concentration enhances its efficacy, reaching 57.14% ± 0.5% at 600 µg/mL. NSEO and CHX 0.2% had a good percentage of hemolysis inhibition; it inhibited 64.70% and 65.88% hemolysis, respectively, as compared to the standard drug aspirin (72.00%) (Fig. 6A). Similar trend was followed by results of ice induced haemolysis method (Fig. 6B).

### Antimicrobial activity

#### Antibacterial and antifungal activity against oral pathogens

**Figure 7:**
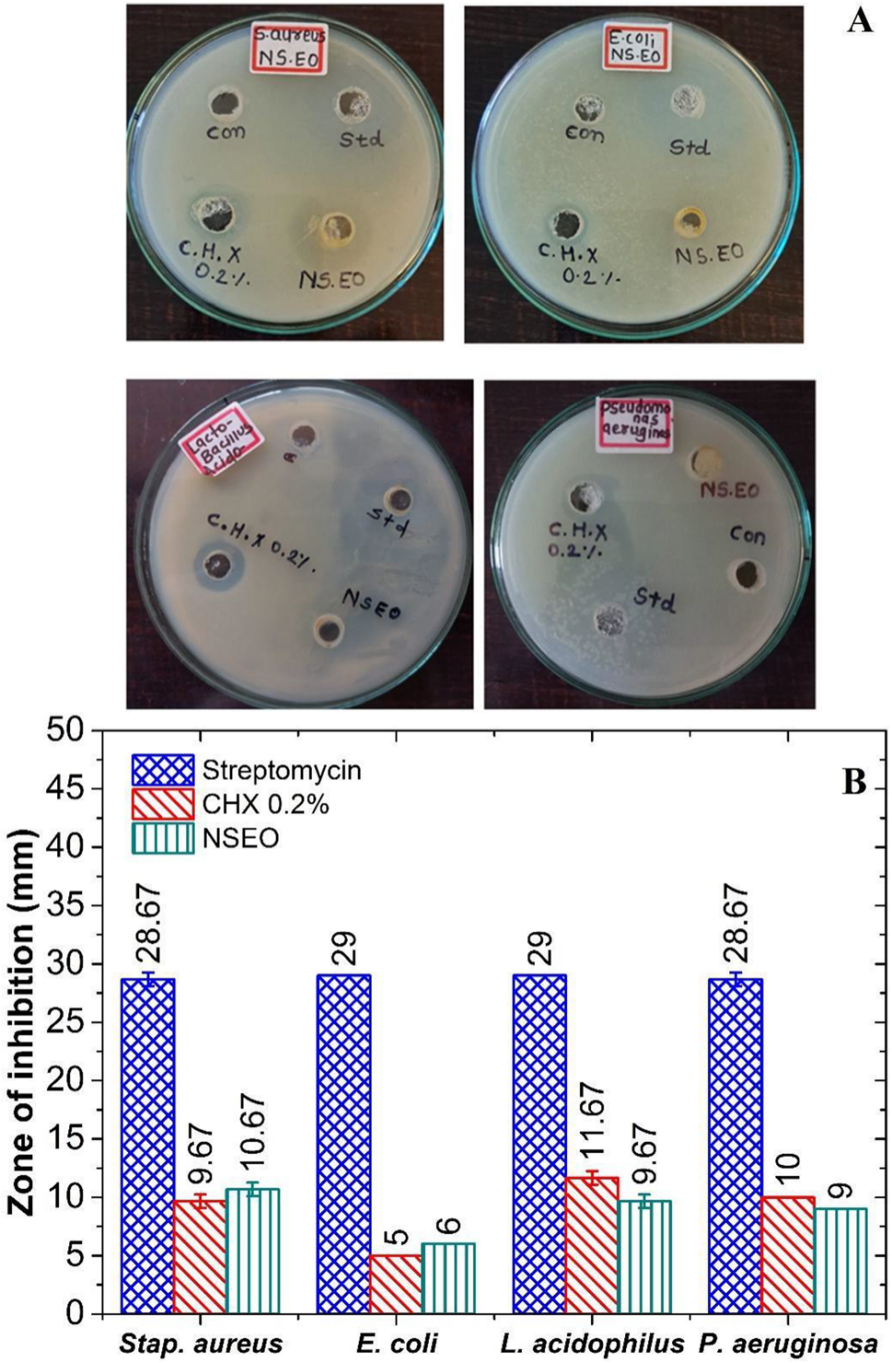
(**A**) photographs depicting zones of inhibition of streptomycin, CHX 0.2%, and NSEO against bacterial strains and (**B**) graphical representation of zone of inhibitions.

Gingivitis and periodontitis are primarily caused by bacteria, typically extending to adjacent supporting tissues, leading to tissue destruction and tooth loss^44,45^. In line with dental and periodontal health, restriction of bacterial development is therefore necessary. The antibacterial profile of NSEO and other various standard compounds (Streptomycin and CHX 0.2%) was determined by calculating inhibition zone against Gram negative (*Pseudomonas aeruginosa* ATCC 27853 and *Escherichia coli* NCIM 2832) and Gram positive (*Lactobacillus acidophilus* ATCC 700396 and *Staphylococcus aureus* NCIM 2654) bacterial strains through well diffusion technique. Compared to the conventional streptomycin, NSEO and CHX 0.2% demonstrated moderate activity. In comparison to streptomycin, the antibacterial activities of NSEO and 0.2% CHX differed significantly (p < 0.05). However, there was no significant difference (p > 0.05) for the antibacterial effects of NSEO and 0.2% CHX against Escherichia coli, Staphylococcus aureus, and Pseudomonas aeruginosa. NSEO exhibited comparatively lesser activity (p < 0.05) against *L. acidophilus* than CHX 0.2% (Fig. 7A,B). For NSEO, highest vulnerable bacterial strain was *Stap. aureus*, whereas the *E coli* was the highest resistant bacterial strain.

**Figure 8:**
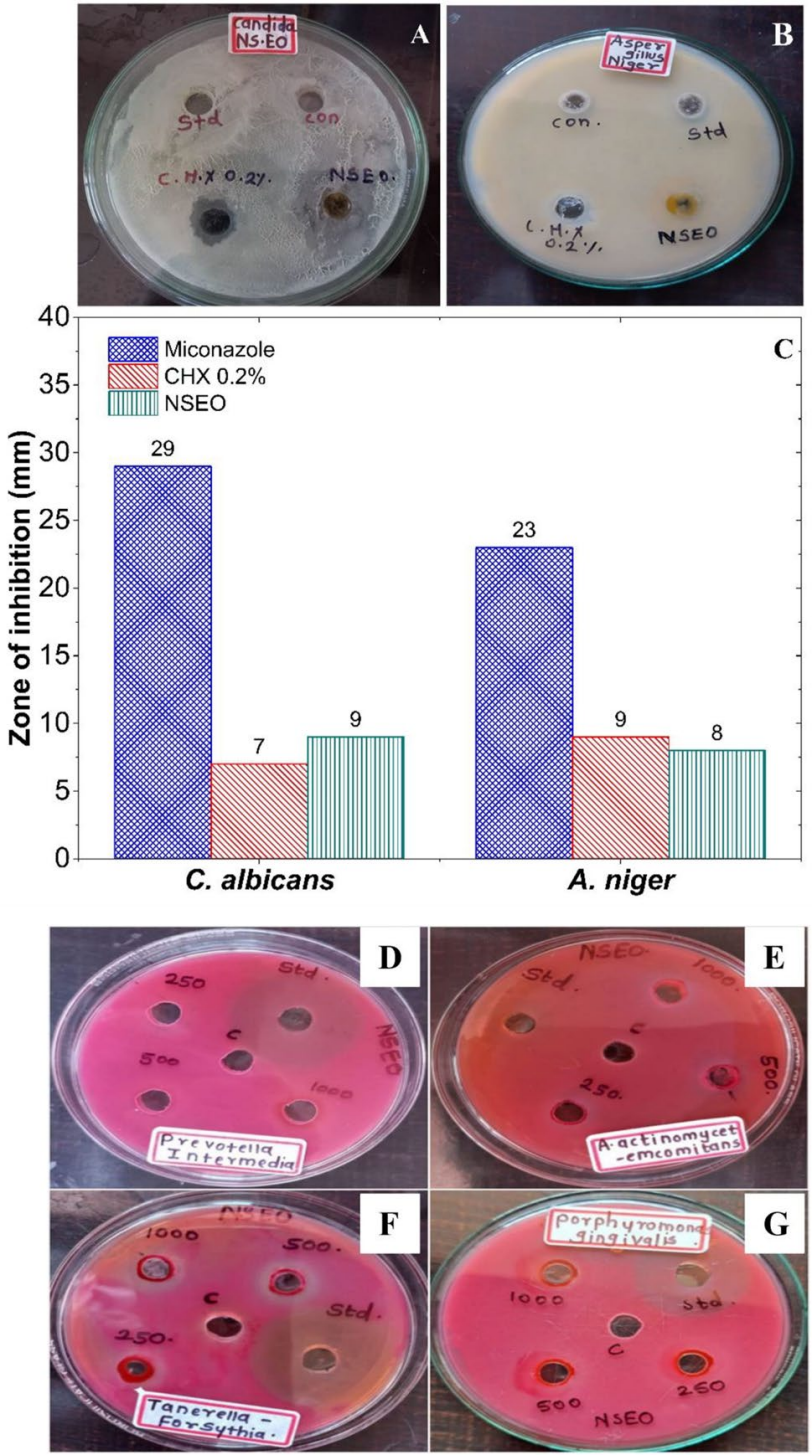
Photographs depicting zones of inhibition of miconazole, CHX 0.2%, and NSEO against (**A**) *C. albicans* and (**B**) *A. niger* (**C**) Graphical representation of obtained zones of inhibitions. Photographs presenting zones of inhibition of metronidazole, CHX 0.2%, and NSEO against (**D**) *P. intermedia* (**E**) *A. actinomycetemcomitans* (**F**) *T. forsythia* and (**G**) *P. gingivalis.*

Moreover, the antifungal activities of CHX 0.2% and NSEO compared to standard miconazole were statistically significant (p < 0.05). In addition, NSEO activity against C. albicans was higher (p < 0.05) in comparison to CHX 0.2% (Fig. 8A). However, antifungal effects between NSEO and CHX 0.2% against *A. niger* were not statistically significant (p > 0.05) (Fig. 8B). Graphical representation of zones of inhibition shown by standard, CHX, and NSEO is shown in Fig. 8C.

#### Antibacterial activity against periodontal pathogens

Compelling evidence underscores the correlation between the abundance and presence of diverse periodontal pathogens, notably *P. gingivalis* and *F. nucleatum*, increased inflammation and pocket depth in periodontitis patients^46^. NSEO was studied for its inhibition potential against periodontal pathogens, *A. actinomycetemcomitans, P. gingivalis, T. forsythia* and *P. intermedia*. Observed zones of inhibition (mm) were measured *[A. actinomycetemcomitans* (15.11 ± 0.15) > *T. forsythia* (13.05 ± 1.76) > *P. gingivalis* > (12.41 ± 0.41), *P. intermedia* (12.12 ± 0.16)] in comparison with Metronidazole (standard), CHX 0.2% as a positive control and distilled water as a negative control (Fig. 8D–G). Among these, *A. actinomycetemcomitans* showed significant change. However, CHX 0.2% in comparison with NSEO has shown statistically significant results for all organisms except *P. gingivalis.*

#### Minimum inhibitory concentration

**Figure 9:**
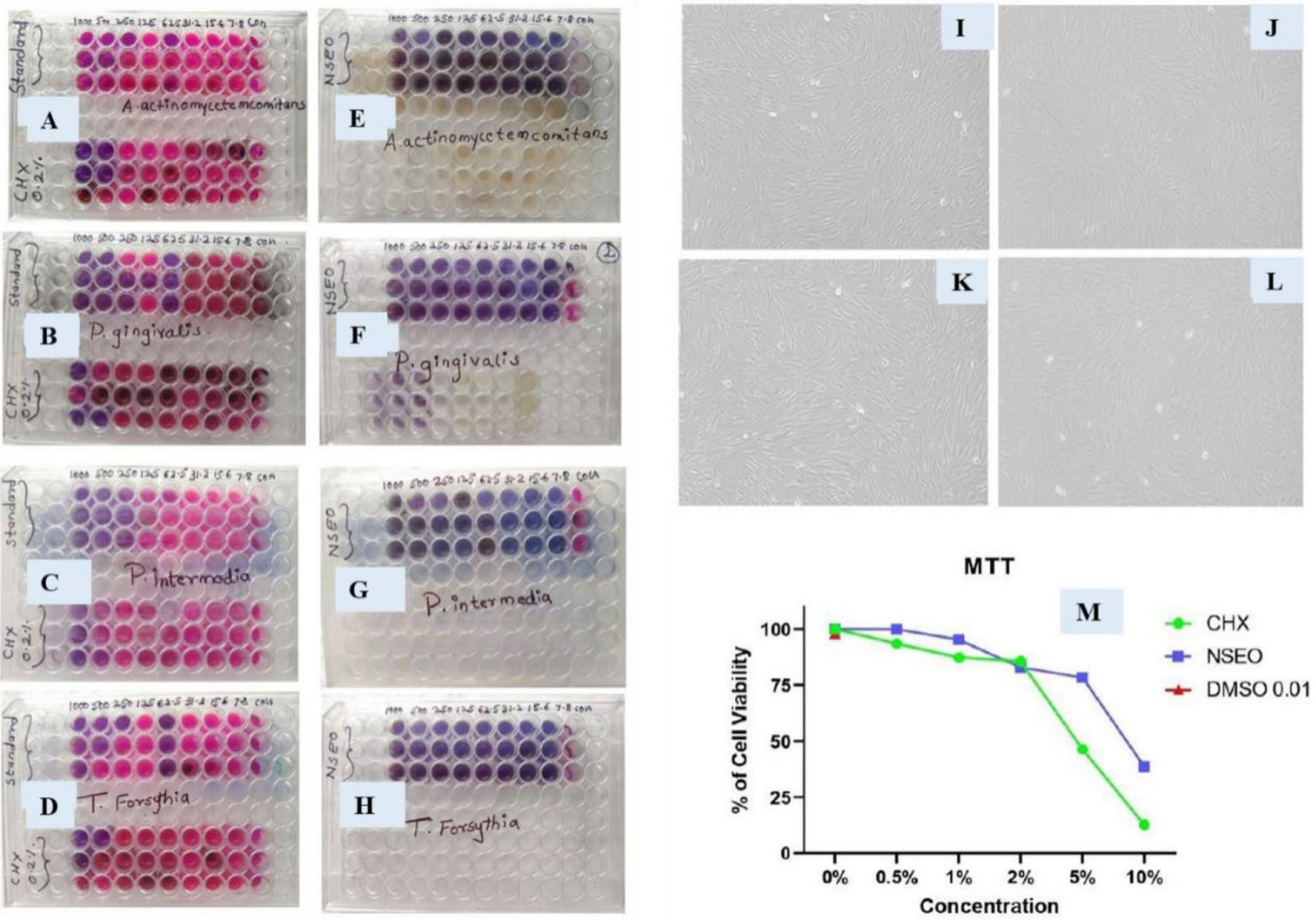
(**A**,**E**) MIC of NSEO for *A. actinomycetemcomitans*, (**B**,**F**) MIC for *P. gingivalis,* (**C**,**G**) MIC for *P. intermedia* (**D**,**H**) MIC for *T. forsythia* (**I**–**L)** Cytotoxicity of NSEO over human gingival fibroblast cell lines. (**M**) Graphical representation of cell viability.

MIC of the NSEO was examined against periodontal pathogens *A. actinomycetemcomitans, P. gingivalis, T. forsythia* and *P. intermedia.* NSEO inhibited the bacterial growth at *A. actinomycetemcomitans *(< 31.2 µg/mL),* P. gingivalis *(31.2 µg/mL),* T. forsythia *(< 31.2 µg/mL) and* P. intermedia *(31.2 µg/mL) as compared to the standard drug Metronidazole (< 7.86 µg/mL) and positive control CHX 0.2% (15.6 µg/mL) at the concentration 7.8–1000 µg/mL (Fig. 9A–D). Notably, NSEO has demonstrated statistically significant effects in each tested organism and concentration, with the most pronounced effects observed particularly at 7.8 and 15.6 µg/mL, highlighting its efficacy.

#### Minimum bactericidal concentration

The MBC of the NSEO was examined to estimate their bactericidal potential against periodontal pathogens *A. actinomycetemcomitans, P. gingivalis, T. forsythia* and *P. intermedia.* NSEO showed MBC at 15.6 µg/mL as compared to standard Metronidazole (7.8 µg/mL) and positive control CHX 0.2%. Whereas the test sample and positive control showed marginal similar change (Fig. 9E–H).

### Cytocompatibility of NSEO over human gingival fibroblast cell lines

NSEO was tested for five concentrations (5, 10, 20, 50, 100 µg/mL by MTT Method to check its cytocompatibility. NSEO revealed that, NSEO has very good cytocompatibility (82%-cell viability) against normal human gingival fibroblast cell line as compared to the positive control CHX 0.2% (Fig. 9I–L). As the concentration increases the percentage of inhibition of normal cells increases slowly and percentage of viability decreases respectively. It indicates that NSEO lightly affect the normal cells at its highest used concentration i.e., at 10% NSEO concentration it inhibits the nearly 20% normal cells (Fig. 9M). As per ISO 10993-514 the material or compound that do not inhibit viability of > 30% of cells are considered cytocompatible^47^. Therefore, it is evident that NSEO has good cytocompatibility as compared to positive control CHX 0.2%.

## Discussion

Plant-based compounds offer significant health benefits and play a crucial role in well-being^48,49^. To understand their pharmacological importance and potential health risks, it's essential to determine the phytochemical contents of therapeutic plants or extracts^50^. The phytochemical analysis of NSEO indicates a diverse array of bioactive compounds, such as phenolics, flavonoids, and terpenoids, known for antioxidant, anti-inflammatory, and antimicrobial properties essential for oral health. These results corroborate previous findings, highlighting NSEO as a valuable source of medicinal phytochemicals^51^. The hydro-distillation process effectively isolated NSEO, exhibiting a pale-yellow hue consistent with the desired brownish-yellow color observed in prior research on NSEO's physicochemical properties^39,52^.

The GCMS/MS analysis of NSEO unveiled a diverse profile of bioactive compounds, such as flavonoids, terpenoids, and fatty acids, renowned for their antioxidant, anti-inflammatory, and antimicrobial effects. These results align with previous studies^53–60^, reinforcing the medicinal potential of NSEO for various health applications.

Phenolic substances in plants are known for their antioxidant properties, crucial for combating oxidative stress implicated in oral inflammatory conditions like periodontal disease and gingivitis^61^. Our study revealed a low total phenolic content (TPC) in NSEO. Guergouri et al. reported TPC in NSEO as 16.67 ± 0.35 µg GAE/g extract^62^, while Singh et al. found it to be 11.47 ± 0.05 (mg GAE/g)^63^. These variations in TPC could be attributed to factors such as sugar, ascorbic acid, or carotenoid concentrations, as well as differences in geographical location and extraction methods^29^. Guergouri et al. found a TFC of 3.83 ± 0.02 (µg QAE/g extract) in black cumin seed oil^62^. Genetic variation and agroclimatic factors affect phytochemical synthesis and concentration^64^.

Assessment of NSEO's antioxidant activity through assays like SOD enzymatic, ABTS + radical scavenging, ferric ion reducing, and DPPH radical scavenging provides valuable insights into its ability to neutralize ROS and protect oral tissues. These findings are significant as antioxidants help prevent oxidative damage to cells and tissues, supporting oral health maintenance and disease prevention. Our DPPH antioxidant assay results surpass those of Mohammed et al.^65^, who reported IC50 values of 1587.3 µg/mL (SFE) and 2308.6 µg/mL (CP) for NSEO. However, Burits et al. found NSEO required 460 µg/mL for 50% inhibition^66^. Sherwani et al. observed 67.33% scavenging activity at 600 µg/mL^67^. Antioxidant activity may be credited to constituents like thymoquinone in NSEO^68^. EO’s diverse antioxidant properties stem from constituents like terpenes and phenolic compounds^69^.

The increased reducing capability of NSEO as evident by FRAC assay, attributed to phenolic components like carvacrol, is complemented by compounds such as alcohols and hydrocarbons, enhancing its antioxidant effectiveness^69^. Our results outperform Mohammed et al., who reported IC50 values of 538.67 ± 12.58 (SFE) and 29.00 ± 54.78 mmol/100 mL (CP) for NSEO^65^.

Oxidative stress can result from increased ROS production or weakened antioxidant defenses. Superoxide dismutase (SOD) is crucial for cellular defense against ROS, converting superoxide radicals to hydrogen peroxide (H_2_O_2_), and then to harmless oxygen and water. Diminished antioxidant enzyme activities likely lead to reduced ROS scavenging, increasing tissue susceptibility to oxidative damage. Conversely, oral administration of NSEO led to a significant increase in SOD enzyme activity^47,70^.

Inflammation plays a critical role in oral diseases like periodontitis and oral mucositis, often marked by tissue protein denaturation. Dental infections may stem from oral bacteria's proteolytic activity, deteriorating periodontal tissue and tissue fluid. Protein degradation in tissue fluid and amino acid fermentation support microbial growth, highlighting the importance of targeting protein denaturation to develop anti-inflammatory drugs^71,72^.

NSEO exhibits potential anti-inflammatory properties by inhibiting protein denaturation and stabilizing red blood cell membranes, which could mitigate tissue damage and promote oral health. Compared to standard chlorhexidine (CHX) 0.2%, NSEO's effects are promising. Discrepancies with existing literature may arise from sample variability, extraction techniques, and experimental conditions. However, our results surpass those of Belal et al., who found a 58.18% inhibition of albumin denaturation at 1000 µg/mL^72^. Additionally, our research outperforms Sherwani et al., who noted a 57.95% reduction in egg albumin denaturation at 600 μg/mL^67^. Our investigation's findings on the HRBC stabilization assay demonstrate concentration-dependent membrane stabilization, suggesting that NSEO ingredients maintain membrane tonicity and prevent HRBC membrane lysis. This could be attributed to the diverse phytochemicals present in NSEO.

The hemolytic activity of oral pathogens *like Porphyromonas gingivalis* contributes to periodontal disease progression. NSEO's inhibition of hemolysis suggests its potential to protect against microbial-induced hemolysis, supporting oral tissue integrity and health. Both NSEO and CHX 0.2% demonstrate anti-hemolytic activity, with NSEO showing notable efficacy. Iqbal et al. reported 65.866 ± 3.066% inhibition of heat-induced erythrocyte hemolysis by NSEO at 500 µg/mL^71^. However, our results fall short of those observed by Sherwani et al., who found N. sativa extract stabilized RBC by 57.86% at 600 µg/mL^67^.

The antimicrobial properties of NSEO are crucial in oral health, where microbial infections contribute significantly to conditions like dental caries, periodontal disease, and oral candidiasis. *E. coli*, *S. aureus*, and *P. aeruginosa* are known pathogens associated with oral infections and can serve as indicators of antimicrobial efficacy in oral care products. Oral pathogenic bacteria including *Lactobacillus acidophilus*, *Aggregatibacter actinomycetemcomitans*, and *Candida albicans* are implicated in the initiation and progression of dental caries and periodontitis.There is a time-dependent colonization of the oral cavity by *Staphylococcus aureus* and *Candida* species in patients with poor oral hygiene. Escherchia coli has been identified as the most common gram negative organism in dental samples. *P. aeruginosa* has been known to be present in dental biofilms associated with dental implants, causing peri-implantitis^44,75–77^.

Understanding NSEO's antimicrobial activity spectrum and selectivity against oral pathogens offers insights into its therapeutic potential. Consistent with previous research^44,45,77^, NSEO shows greater vulnerability among Gram-positive bacteria. The varying susceptibility may stem from differences in cell wall composition, with Gram-positive bacteria having lower lipid content^78,79^. Gram-negative bacteria possess hydrophilic polysaccharide chains in their outer membranes as defence against hydrophobic essential oils^80^. However, literature presents contradictions, suggesting equal vulnerability of both Gram-positive and Gram-negative bacteria to essential oils^81^. Some suggest Gram-negative bacteria are more susceptible to essential oils than Gram-positive bacteria^82^, but most studies align with our findings.

In our study, measured zones of inhibition (mm) were: *A. actinomycetemcomitans* (15.11 ± 0.15 mm) > *T. forsythia* (13.05 ± 1.76 mm) > *P. gingivalis* (12.41 ± 0.41 mm) > P*. intermedia* (12.12 ± 0.16 mm). Harzallah et al.^83^ found significant antibacterial efficacy of NSEO (zone of inhibition: 13.5–15.5 mm). Another study reported 19.3 mm inhibition against *E. coli*^84^. Similarly, significant inhibition zones were documented: 19 mm against *S. aureus* (ATCC 103207) and 21 mm against *E. coli* (ATCC 12079)^85^. Zouirech et al. reported 38.67 mm inhibition against *E. coli* K12^69^.

The robust antibacterial capacity of NSEO underscores its potential as an alternative therapeutic approach against bacterial resistance, distinct from conventional antibiotics. Our findings align with prior literature, highlighting NSEO's efficacy against specific bacterial strains. This antimicrobial effectiveness is attributed to the precise arrangement of the hydroxyl group within the phenolic structure of these compounds. They alter permeability and induce leakage of intracellular components by selectively binding to membrane-bound proteins' amine and hydroxylamine groups in bacterial cells^86,87^.

The hydrophobic nature of NSEO facilitates lipid separation between bacterial cell membranes and mitochondria, potentially contributing to its antibacterial properties. This disrupts cell structures, enhancing penetration. Significant leakage of bacterial cells or escape of vital chemicals and ions may lead to bacterial cell death^84^.

Humans have had oral fungal infections for thousands of years. *Candida* species are the most prevalent cause of oral fungal infections, although other fungal genera are also frequently responsible, such as *Aspergillus*, *Geotrichum, Rhodotorula, Malassezia*, *Cladosporium, *etc.^40,88,88^. To combat various fungal diseases, compounds with broad-spectrum antifungal activity are highly desirable. NSEO's increased efficacy against Candida albicans indicates potential applications in antifungal therapy. The breakdown, modification, and prevention of cell wall construction could all contribute to NSEO's antifungal action. EO constituents may act as antifungal agents by accumulating in the lipophilic hydrocarbon molecules of the cell's lipid bilayer^40^. The significant antifungal activity against *C. albicans* can be attributed to thymoquinone, consistent with findings from previous studies^89,90^. Our study's results fall short of those achieved by Zouirech et al.^69^, who reported a 42 mm zone of inhibition against *C. albicans* with NSEO, compared to our modest 9 mm. However, unlike Zouirech et al., who did not observe any activity against *A. niger*, our study successfully achieved activity against this strain.

The anti-bacterial activity against periodontal pathogens in this study aligns with previous literature. Tawfig^91^ reported significant bactericidal activity of *N. sativa* against *P. gingivalis*, with mean zone of inhibitions of 6.2 ± 0.2 mm and 8.4 ± 0.3 mm at concentrations of 25 and 50 mg/mL, respectively. Additionally, a mouth rinse containing *N. sativa* demonstrated promising effects against both *P. gingivalis* and *A. actinomycetemcomitans*, with mean inhibition zone diameters of 25.141 mm and 25.1 mm, respectively^46^. These reports underscore the effectiveness of NSEO against periodontal pathogens, attributed to the presence of thymoquinone. Thymoquinone has been shown to facilitate bone repair in defects infected with *P. gingivalis*, enhance angiogenesis rates, and reduce the duration required for bone repair^92^. Furthermore, 0.2% Thymoquinone exhibited sensitivity against *P. gingivalis*, *A. actinomycetemcomitans*, and *P. intermedia*^93^, and demonstrated potent antibacterial effects and reduced virulence properties of both *F. nucleatum* and *P. gingivalis*^94^. However, the antibacterial role of NSEO against important periodontal pathogens remains limited in literature.

The absence of cytotoxicity to normal fibroblast cells underscores the safety profile of NSEO, making it a promising natural alternative in oral care. Our study, in line with Juniperus excelsa M. Bieb essential oil, demonstrated superior cytocompatibility with HGF compared to CHX 0.05%^95^. However, literature on NSEO's cytocompatibility with gingival fibroblasts remains limited.

While this study sheds light on NSEO's therapeutic potential in oral health, further research is needed to understand its mechanisms, optimize dosages, and assess long-term safety and efficacy. Future studies should explore tailored formulations and delivery systems. In summary, our comprehensive evaluation underscores NSEO's significant potential as a natural agent for promoting oral health and combating oral inflammatory disorders. Targeting multiple disease aspects, NSEO offers a promising avenue for novel oral care interventions with enhanced efficacy and safety.

## Conclusions

An increasingly concerning issue in medical research is the rising bacterial and fungal resistance to many synthetic antimicrobial medicines. Ethnopharmacological studies have demonstrated that plants harbor biologically active antibacterial substances, with *N. sativa* being a well-recognized evidence-based herbal remedy due to its remarkable therapeutic properties. In this study, the hydrodistillation technique facilitated the isolation of NSEO, with phytochemical analysis confirming the presence of steroids, triterpenoids, alkaloids, flavonoids, phenols, proteins, and tannins. NSEO exhibited notable anti-inflammatory, antibacterial, and antifungal effects, particularly against periodontal bacteria, an area with limited information in previous studies. Additionally, our findings revealed a cytoprotective effect of the essential oil on human gingival fibroblast cell lines. These encouraging outcomes, in reducing inflammation and halting the spread of oral infections, open the door for the development of novel treatments for periodontitis and other inflammatory oral diseases. However, further in vivo studies are necessary to substantiate the current results.

## Data Availability

The datasets used and/or analysed during the current study are available from the corresponding author on reasonable request.
